# Hepatoprotective role of liver fatty acid binding protein in acetaminophen induced toxicity

**DOI:** 10.1186/1471-230X-14-44

**Published:** 2014-03-10

**Authors:** Yu Gong, Guqi Wang, Yuewen Gong, Jing Yan, Yufei Chen, Frank J Burczynski

**Affiliations:** 1 Faculty of Pharmacy, University of Manitoba, 750 McDermot Avenue, Winnipeg, MB R3E 0T5, Canada; 2 Department of Pharmacology and Therapeutics, Faculty of Medicine, University of Manitoba, Winnipeg, MB, Canada; 3 Liver-Biliary-Pancreatic Center, Carolinas Medical Center Charlotte, Charlotte, NC 28232-2861, USA

**Keywords:** FABP1, Acetaminophen, Oxidative stress, Liver, Apoptosis

## Abstract

**Background:**

FABP1 has been reported to possess strong antioxidant properties. Upon successful transfection of the Chang cell line, which has undetectable FABP1 mRNA levels, with human FABP1 cDNA, the Chang cells were shown to express FABP1. Using the transfected and control (normal) Chang cells and subjecting them to oxidative stress, transfected cells were reported to be associated with enhanced cell viability. This study extends those observations by investigating the effect of FABP1 on acetaminophen (AAP)-induced hepatotoxicity. We hypothesized that presence of FABP1 would enhance cell viability compared to control cells (vector transfected cells).

**Methods:**

Following AAP treatment of Chang FABP1 transfected and control cells, cell viability, oxidative stress, and apoptosis were evaluated using lactate dehydrogenase (LDH) release, the fluorescent probe DCF, and Bax expression, respectively.

**Results:**

FABP1 cDNA transfected cells showed greater resistance against AAP toxicity than vector transfected cells. Significantly lower LDH levels (p < 0.05) were observed as were lower DCF fluorescence intensity (p < 0.05) in FABP1 cDNA transfected cells compared to vector transfected cells. FABP1 expression also attenuated the expression of Bax following AAP induced toxicity.

**Conclusion:**

FABP1 attenuated AAP-induced toxicity and may be considered a cytoprotective agent in this *in vitro* model of drug induced oxidative stress.

## Background

Fatty acid binding protein (FABP) was first discovered in 1969 by Levi et al. [1]. Liver fatty acid binding protein (FABP1), named after the tissue in which it was first identified, is approximately a 14–15 kDa protein mainly present in the cytoplasm of hepatocytes but expressed in many other tissues [2,3]. FABP1’s main function is thought to be the intracellular transport of lipophilic substrates such as long chain fatty acids. FABP1 also interacts indirectly with the peroxisome proliferator-activated receptor alpha (PPARα), which is a key regulator of lipid homeostasis in hepatocytes and a target for fatty acids and hypolipidemic drugs, by transporting PPARα agonists to its site. Thus, FABP1 acts as a cytosolic gateway by directing PPAR ligands to the nucleus [4].

A new antioxidant property of FABP1 has recently been uncovered [5,6]. In its primary structure FABP1 contains seven methionine and one-cysteine amino acids. These groups are regarded as cellular scavengers of activated xenobiotics and are involved in trapping of free radicals [7]. Thus, in this study we used an acetaminophen induced toxicity model in cultured cells to assess the antioxidant function of FABP1.

Acetaminophen (AAP), 4-hydroxyacetanilide, is a widely utilized drug known for its analgesic and antipyretic properties. When used at therapeutic levels it is safe, however, an acute or cumulative overdose can cause severe liver injury with the potential of liver failure [8]. At therapeutic doses, AAP is primarily detoxified by glucuronidation and sulfation with a small fraction metabolized by cytochrome P-450-dependent mixed function oxidase system to a highly reactive N-acetyl-p-benzo-quinonemine (NAPQ1) metabolite [9]. The metabolite reacts with glutathione (GSH) spontaneously or is catalyzed by glutathione-S-transferases to form a GSH-adduct which is mainly excreted into bile through Mrp2 without significant toxicity [10]. After an AAP overdose, however, glucuronidation and sulfation are insufficient to detoxify AAP. A large fraction of the drug becomes available for metabolism by cytochrome P450, leading to a rapid depletion of hepatic GSH levels. Once GSH is exhausted, any remaining NAPQI formed will react with alternative targets, in particular cellular proteins [11]. In addition to NAPQI, other cellular effects of AAP toxicity further exacerbates cellular oxidative stress which in turn contributes to the cell injury process [12].

The present study investigates the hepatoprotective effect of FABP1 in acetaminophen-induced toxicity using the Chang cell line. Chang cells were originally thought to be derived from normal liver tissue, but subsequently found to have been established via HeLa cell contamination. The rationale for using this cell line, however, rests in the fact that these cells were shown to be devoid FABP1 [5], yet have the metabolic enzymes responsible for metabolizing AAP to the NAPQ1 reactive species [12]. Thus, it is an ideal cell line to study the biological and bioprotective effects of FABP1.

## Methods

### Materials

Dulbecco’s modified Eagle’s medium (DMEM), sodium pyruvate, penicillin, streptomycin, Geneticin (G-418) and sodium pyruvate were purchased from GIBCO/BRL (Life Technology, Burlington, ON). Acetaminophen, calf serum and β- nicotinamide adenine di-nucleotide, reduced form of β-NADH, were purchased from Sigma (Sigma, Co., St. Louis, MO). The cellular toxicity assay kit (WST-1) was purchased from Roche (Roche Diagonstics GmbH, Mannheim, Germany), anti-Bax (N-20) monoclonal antibody was from Santa Cruz Biotechnology, Inc., horseradish peroxidase conjugated anti-mouse lgG (sheep) was from Amersham biosciences, anti-rabbit lgG horseradish peroxidise linked whole antibody was from GE healthcare, and rainbow molecular weight marker was purchased from Invitrogen. FABP1 polyclonal antibody was generated in our laboratory.

### Methods

#### Cell cultures

Chang cells were obtained from American Type Culture Collection (Manassas, VA, USA). Vector and FABP1 cDNA transfected Chang cells [5] were grown in DMEM supplemented with 100 U of penicillin/ml, 100 μg streptomycin/ml and 10% calf serum, in a humidified 37°C incubator with an atmosphere of 95% air and 5% CO_2_. The transfected Chang cells were maintained in the presence of G418 (Geneticin) at a concentration of 200 mg/L.

Cells were seeded at a density of 1 × 10^4^ cells/well (for WST-1 and DCF assay, 96-well plates), 1 × 10^5^ cells/well (for LDH assay, 6-well plates) and 1 × 10^6^ cells/well (for Western blot, 60 mm dishes) in DMEM medium and incubated overnight for adherence. The next day cell cultures were washed once with warm phosphate-buffered saline (PBS) and incubated with AAP for 3, 6, 12, and 24 hrs at 37°C in a humidified incubator in an atmosphere of 95% air and 5% CO_2_. At the end of the incubation period, cells were prepared for the various assays described below.

#### Cell viability

The cell proliferation reagent WST-1 is based on the metabolic activity of viable cells. WST-1 (10 μl/well) was added to cells that had already been treated with AAP (0 mM, 0.5 mM, 1 mM, 5 mM, 10 mM, and 20 mM) for 3, 6, 12, and 24 hrs, followed by 3 hrs incubation with WST-1. Cell viability was measured using a spectra Max 190 (Molecular Devices) plate reader at 440 nm.

The release of lactate dehydrogenase (LDH) from cells during the incubation period with AAP was used to determine cell damage. Briefly, after treatment with 10 mM AAP for 3, 6, 12 and 24 hrs, LDH activity in the supernatant was determined as follows: a 10% (v/v) 2.5 mM NADH was mixed with 10% (v/v) 25 mM pyruvate in a Tris-KCl buffer (50:150 mM, PH = 7.4) on the day of the assay and equilibrated to 25°C in a water bath prior to use. Aliquots (50 μl) of the culture supernatants (containing LDH) were added to a quartz cuvette that contained 1000 μl substrate solution and then measured in UV-visible recording spectrometer (UV-160). Change in absorbance at 340 nm was directly proportional to LDH activity in the supernatant samples.

#### DCF measurements

Fluorescence intensity of DCF from reactive oxygen species (ROS) formed in cell cultures was assessed using a Wallac 1420 multilable counter (Perkin Elmer). A 10 mM (4.87 mg/ml) stock solution of 2,7- dichlorofluorescein diacetate (H_2_DCFDA) was prepared daily in ethanol and stored at – 20°C until required and diluted to 1 mM with PBS prior to each study. Following incubation with AAP, cells were washed twice with Ca^++^/Mg^++^-PBS and incubated with 100 μM 2,7-dichlorofluorescin diacetate (DCFH_2_-DA) at room temperature in the dark for 30 min. Mean of fluorescence intensity was calculated from 4-wells of control and drug-treated cells from six separate experiments.

#### Western blot

After AAP treatment, protein extracts were prepared from cell cultures using RIPA Buffer (50 mM Tris–HCl pH 7.4, 150 mM NaCl, 1 mM PMSF, 1 mM EDTA, 5 μg/ml Aprotinin, 5 μg/mL Leupeptin, 1% Triton x-100, 1% Sodium deoxycholate, 0.1% SDS). Briefly, cell pellets (1 × 10^6^) were washed once with ice-cold PBS and incubated with 1 ml RIPA buffer on ice for 20 min, vortexing 2–3 times. The lysate was centrifuged for 5 min at 4°C at 20,000 *g* in microfuge tubes. Supernatants were transferred to clean tubes. Protein concentration was measured using the BCA Protein Assay. Samples were stored at −80°C until required. The optical density values of each target protein band were determined using NIH Imaging software.

#### Statistical analyses

Statistical analyses of the treatment groups were carried out by *t* test (unpaired) where 2 groups were compared while a two-way ANOVA was used for multiple comparisons. Statistical differences with *P* values <0.05 were taken as significant.

## Results

### Cellular viability

**Figure 1:**
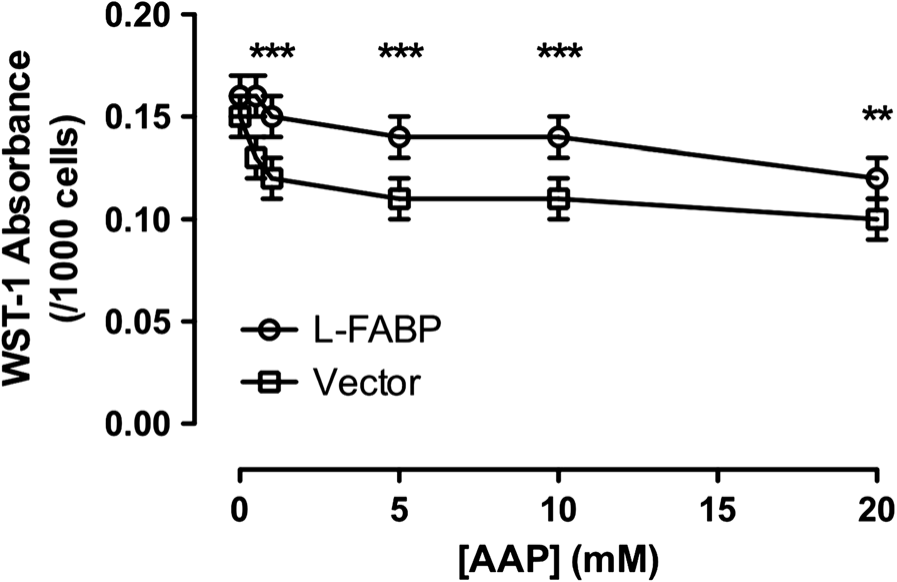
**Cytotoxicity of vector and FABP1 transfected cells exposed to various AAP concentrations for 3 hrs as measured by WST-1.** The two groups of cells were treated with 0, 0.5, 1, 5, 10, 20 mM concentrations of AAP for 3 hrs. Data show mean ± SE; n = 6; **p < 0.01 (for 20 mM AAP), ***p < 0.001 (for 0.5 mM, 1.0 mM, 5 mM, and 10 mM AAP) between FABP1 cDNA and vector transfected cells during the same time period.

Cytotoxicity induced by AAP in vector and FABP1 cDNA transfected cells using the cell proliferation reagent WST-1 is shown in Figure 1. AAP induced a dose-dependent cytotoxicity in both vector and FABP1 cDNA transfected cells. The FABP1 cDNA transfected cells, however, showed lower cytotoxicity then vector-transfected cells following 3 hrs of AAP treatment. Drug exposure times of 6 hrs, 12 hrs and 24 hrs, were also investigated with results similar to those shown for 3 hrs (data not shown). Vector transfected Chang cells showed a 15%, 19%, 23%, 27%, and 37% decrease in viability at concentrations of 0.5 mM, 1 mM, 5 mM, 10 mM, and 20 mM AAP, respectively; while cell viability in the FABP1 cDNA transfected cells showed a 2%, 6%, 14%, 17%, and 23% reduction at the same concentrations, suggesting that FABP1 might have a cytoprotective role against AAP induced cytotoxicity. Comparing the FABP1 cDNA transfected cells to the vector transfected cells at AAP concentrations of 0.5 mM, 1.0 mM, 5 mM, and 10 mM showed highly significant differences at the p < 0.001 level, while an AAP concentration of 20 mM was significant at the p < 0.01 level. There was no statistical difference between the two cell types in the absence of AAP.

### ROS levels

**Figure 2:**
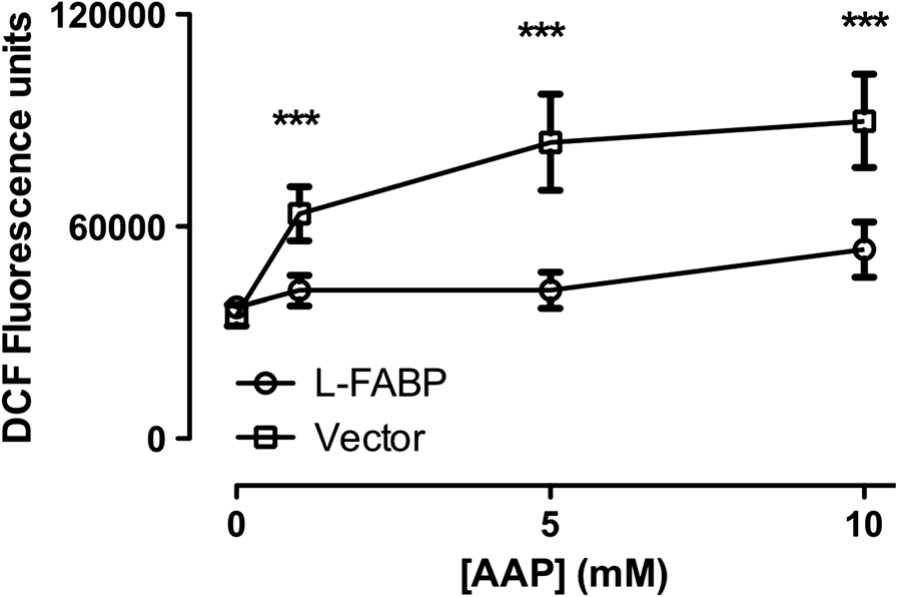
**DCF fluorescence intensity of vector and FABP1 cDNA transfected cells in the presence of 1 mM, 5 mM, 10 mM AAP for 3 hours.** Cells were cultured in black 96-well plates. After AAP treatment H2DCFDA (1 mM) was loaded into wells for 30 minutes. Cellular fluorescence in each well was measured and immediately recorded. Date represent mean ± SEM; n = 6; ***p < 0.001 between FABP1 cDNA transfected and vector transfected cells during the same time period.

Oxidative stress is one of major causes of AAP induced cell injury. As a measure of reactive oxygen species (ROS) we used the ROS-sensitive probe 2,7-dichloro-fluorescein diacetate (H_2_DCFDA) to monitor AAP induced cellular oxidative stress. Figure 2 shows DCF fluorescence results from experiments where vector and FABP1 cDNA transfected cells were incubated for 3 hrs with 1 mM, 5 mM, and 10 mM AAP. Cells incubated with AAP for 6 hrs showed similar results (data not shown). AAP induced a dose-dependent oxidative stress in both vector-transfected cells and FABP1 cDNA transfected cells. DCF fluorescence intensity in FABP1 cDNA transfected cells was, however, significantly reduced (p < 0.05) compared to vector transfected cells at each AAP concentration (Figure 2). Compared to vector transfected cells, DCF fluorescence intensity of FABP1 cDNA transfected cells decreased by 35% ± 5% at 1 mM AAP treatment; 39% ± 5% at 5 mM AAP treatment; and 46% ± 5% at 10 mM AAP treatment (p < 0.001).

### LDH levels

**Figure 3:**
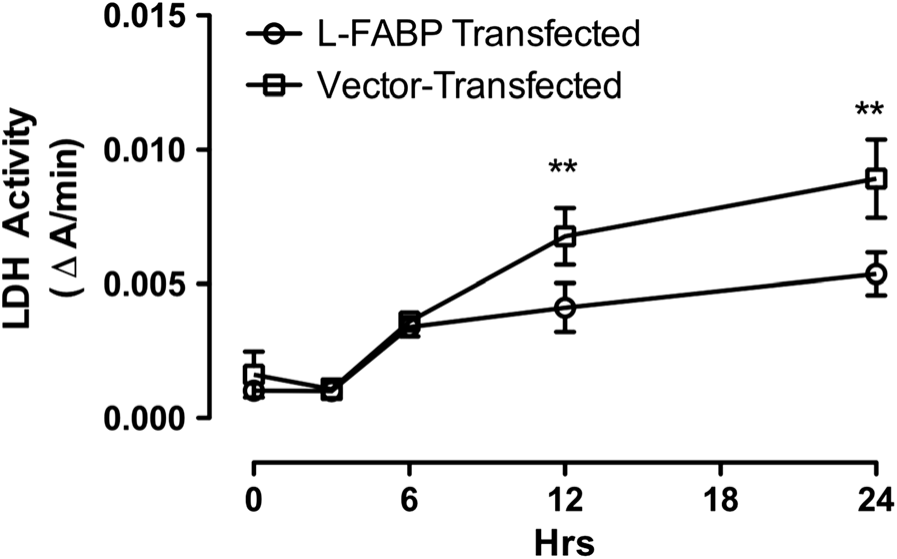
**LDH release from FABP1 cDNA and vector transfected cells subjected to 10 mM AAP treatment for 3 hrs, 6 hrs, 12 hrs, and 24 hrs.** Negative control experiments were performed by incubating cells with no serum DMEM for 24 hrs. LDH absorbance was measured at 340 nm and expressed as the rate of absorbance change per minute. Data represent mean ± SEM (n = 4), **p < 0.01 LDH activity of FABP1 cDNA transfected cells compared with vector transfected cells during the same time period.

The protective effect of FABP1 in AAP induced liver failure was assessed by detecting cellular LDH release. Release of LDH into culture supernatant correlates with reduced cell membrane integrity and cell viability. As shown in Figure 3, a statistical decrease (p < 0.01) in supernatant LDH activity was found in FABP1 cDNA transfected cells only after 12 and 24 hrs of 10 mM AAP treatment compared with vector transfected cells. No statistical difference was observed in the 3 hrs AAP treatment group compared to the control group, suggesting that LDH release may be a late event in AAP induced cell injury that is preventable by FABP1.

### Bax and FABP1 levels

**Figure 4:**
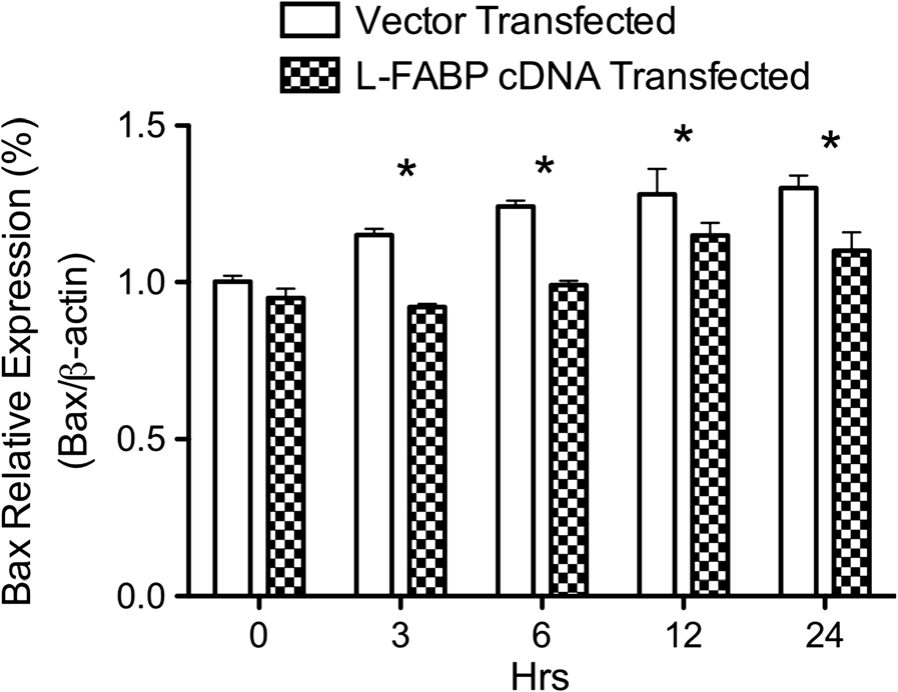
**Expressions of Bax in FABP1 cDNA and vector transfected cells after being treated with 10 mM AAP for 0, 3, 6, 12, and 24 hrs.** β-Actin served as the loading control (n = 6, mean ± SEM, *p < 0.05).

Western blot analysis was used to detect the expression of the pro-apoptotic protein Bax in cells following 0 hrs, 3 hrs, 6 hrs, 12 hrs, and 24 hrs incubation with 10 mM AAP. FABP1 cDNA transfected cells had lower levels of Bax then vector transfected cells at each time period (p < 0.05; see Figure 4). Bax level in the vector-transfected cells started to increase following 3 hrs incubation with AAP and peaked at 12 hrs. Bax level in the FABP1 cDNA transfected cells remained constant until 12 hrs when the Bax level statistically increased from the 6 hrs level (p < 0.05).

**Figure 5:**
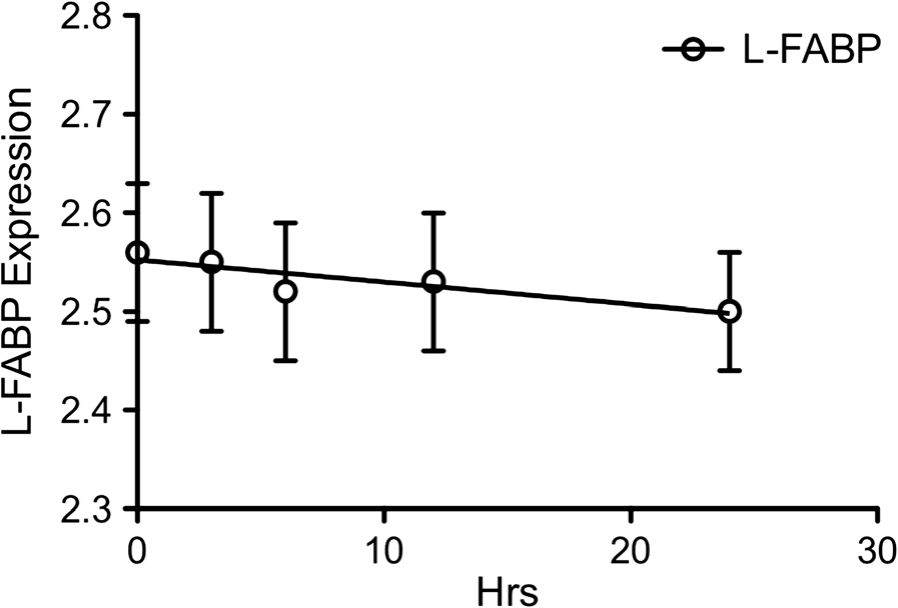
Expressions of FABP1 in FABP1 cDNA transfected cells after being treated with 10 mM AAP for 0, 3, 6, 12, and 24 hrs (n = 6, mean ± SEM).

To verify the role of FABP1 in AAP induced toxicity, FABP1 expression was analysed in each group. As expected there was no FABP1 present in the vector-transfected group while the FABP1 cDNA transfected showed presence of the protein (Figure 5). The FABP1 level decreased over time in the AAP treated group. The slope of the regression line was significantly different from zero (p < 0.05), suggesting that either the AAP toxicity damaged transcriptional/translational events or cells were made leaky such that the FABP1 was able to efflux from cells.

## Discussion

Acetaminophen (AAP) is an effective over the counter medication for relief of minor pain and fever. A small fraction of the AAP dose is metabolized by CYP 2E1 to a highly reactive N-acetyl-p-benzoquinone imine (NAPQI) metabolite. This metabolite is inactivated by glutathione or GSH within the liver. However, if the concentration of NAPQI is high, liver damage can occur. In severe cases liver failure and death occur. Despite its widespread use, the mechanism of AAP’s hepatocellular injury is still being investigated. Many studies have reported impaired mitochondrial respiration [13,14], depletion of hepatocellular ATP levels [15,16], opening of the mitochondrial membrane permeability transition pore [17], and increased levels of glutathione disulfide (GSSG) or the ratio of GSSG:GSH, suggesting the involvement of an oxidant stress following AAP overdose [15,18]. In response to high levels of ROS the nascent hepatocyte antioxidants may not provide sufficient capacity to inactivate them, other antioxidant defence systems are expected to take effect. FABP1, with its high affinity and capacity to bind lipophilic oxidative products [19,20], is a likely candidate for further protecting hepatocytes from ROS.

Our previous work showed that Chang cells were devoid of FABP1. Transfecting the Chang cell line with FABP1 cDNA gave us an opportunity to study this protein in different models of oxidative stress. Using a hypoxia/reoxygenation as well as a H_2_O_2_ induced oxidative stress models [5], we reported that while FABP1 cDNA transfected cells had the same complement of intracellular antioxidant enzymes as the vector transfected cell line, FABP1 cDNA transfected cells were associated with much less ROS levels, suggesting that FABP1 is somehow involved in inactivating free radicals. Yan et al. [21] investigated the mechanism for the antioxidant protective function of FABP1. Rat FABP1 is known to have seven methionine groups in positions 1, 19, 22, 74, 85, 91, and 113, as well as one cysteine group in position 69. Methionine and cysteine are known to react with ROS. Using a recombinant form of rat FABP1 that was cultured in *E. coli*, isolated and purified, the group showed that indeed the methionine groups of FABP1 were associated with reactive oxygen as assessed by MALDI-TOF. Moreover, FABP1 was shown to react with free radicals in both hydrophilic and lipophilic domains of the cell. Using AAPH as a hydrophilic free radical generator the group determined that methionine 1, 19, 22, 91, and 113 were oxidized. In the lipophilic environment, using AMVN as the lipophilic free radical generator, methionine 1, 19, 22, and 113 were only reactive. Methionine 74 and 85 were unreactive in both systems suggesting that these groups may be buried deep within the binding site of FABP1 while the other reactive groups are surface exposed. Interestingly, methionine 91 did not react with any free radicals in the lipophilic domain. This suggested that FABP1 might orientate itself to the membrane allowing other methionine groups access to react with free radicals at the membrane surface. FABP interaction with membrane surfaces has been suggested to occur with FABP2 (intestinal fatty acid binding protein) but not with FABP1 [22].

In this study we investigated the role of FABP1 in drug-induced liver damage. The data show a rapid onset of an intracellular oxidative stress as early as 3 hrs in cultured cells using 2,7-dichlorofluorescein diacetate (H_2_DCFDA) as a marker of intracellular free radical levels. There was a dose-dependent increase in released ROS induced by AAP with DCF fluorescence intensities being significantly lower in FABP1 cDNA transfected cells compared to the vector transfected cells (Figure 2). AAP induced oxidative stress was studied by other groups such as Bajt et al. [23] who showed that a greater than 10 fold increase in ROS levels between 3.5 and 12.5 hrs resulted following 5 mM AAP treatment in cultured murine hepatocytes. Pretreatment of hepatocytes with 20 mM N-acetylcysteine was shown to enhance cellular glutathione content and suppressed the AAP-induced decrease in cell viability. Bajt concluded that AAP-induced oxidant stress precedes cell necrosis and the oxidant stress is involved in the propagation of cell injury.

The effect of FABP1 on AAP-induced cell damage was assessed using the WST-1 assay in our study, which depends on mitochondrial respiration [24,25]. Results from this assay indicated a substantial functional deterioration of hepatocytes following AAP treatment. Similar to the DCF assay, results showed that the cytotoxicity induced by AAP was dose-dependent and statistical differences were observed between FABP1 cDNA and vector transfected cells following 3 hrs drug treatment (Figure 1). Thus, FABP1 protects cellular mitochondrial function in some way. One possibility is by inactivating cytosolic free radicals. These reactive species are not able to interact with the mitochondrial membrane, thus, preserving cellular function. AAP also caused a progressive release of LDH into the culture media, showing cellular damage. The presence of FABP1 attenuated this increase but only at the latter stages of AAP induced cell injury since LDH release only became statistically significant after 12 hrs of treatment. As well as being present in the cytosol, FABP1 is also present in the mitochondria. It is likely that FABP1 plays an early protective role in AAP induced mitochondrial impairment through scavenging free radicals within the mitochondria itself as well as in the cytosol.

The mechanism of AAP-induced cell death is not completely understood. Of the events leading to apoptotic and/or necrotic cell death, Bax seems to be central for the mitochondrial-dependent mechanisms. Bax is a pro-apoptotic Bcl-2 family member that plays a pivotal role in the formation of the mitochondrial permeability pore. Higher levels of Bax are associated with changes in the outer membrane permeability, which are responsible for changes in the inner mitochondrial membrane that leads to disruption in cell function (e.g., membrane potential, cell swelling, leakage). Deletion of Bax has been shown to be associated with dramatic reduction in necrotic injury during myocardial infraction [26]. Thus, Bax may modulate necrosis through mechanisms that are distinct from apoptosis [26,27]. In our study, decreased Bax levels were seen in the AAP treated FABP1 cDNA transfected group at all time points (Figure 4). In the vector-transfected groups the Bax level started to statistically increase at 3 hrs and peaked at 12 hrs. This was not seen in the FABP1 cDNA transfected cells. In the FABP1 cDNA transfected group the Bax level increased only after 12 hr incubation with AAP. Thus, FABP1 shows a higher protective ability at the early time points (3 and 6 hrs) by preventing the increase in Bax level. At the late time points (12 and 24 hrs), Bax level in the FABP1 cDNA transfected group was lower than in the vector-transfected group but still higher than at the 6 hrs FABP1 cDNA transfected group. FABP1 levels, however, were also declining during the late time points. A rationale explanation for the declining FABP1 levels is not apparent. Studies aimed at assessing the time course of FABP1 mRNA activity would be necessary to explain the decrease in FABP1 levels. Presence of necrosis may be a possibility that leads to the release of FABP1. Nevertheless a strong correlation exists between FABP1 and Bax levels, showing that FABP1 prevents cell death through a reduction in Bax activity. Although our work cannot discern whether the higher (or lower) levels of Bax is associated with Bax translocation to the mitochondria, it is reasonable to speculate that the increased levels would lead to increased translocation. Bajt had earlier reported that following 300 mg/Kg intraperitoneal AAP injection to C57BL/6 mice, Bax translocation from the cytosol to mitochondria occurs as early as 1 hr [28]. Thus, FABP1 likely protects cells against AAP hepatotoxicity during the early time periods of hepatocyte injury.

LDH release is indicative of cell death. Membranes become leaky through a variety of processes including that of oxidative damage. Since release of LDH in our study reached statistical significance only after 12 hrs of AAP treatment, this showed that although earlier time points were associated increased Bax level, later time points involved necrosis. As stated above apoptosis and necrosis are not completely independent processes [29]. These two processes share a common mitochondrial permeability transition (MPT) pathway. When the MPT occurs abruptly, ATPase becomes activated which depletes ATP levels leading to membrane rupture and oncotic necrosis. However, the ATP level can remain constant (baseline condition) when the MPT proceeds relatively slowly or the ATPase is inhibited by glycolysis or oligomycin. Under these conditions, necrosis can be blocked and apoptosis occurs. At any time, ATP depletion can supervene to cause secondary necrosis. A new term, necrapoptosis has been introduced to describe a death process that begins with death signals or toxic stress, proceeds by shared pathways, but results in either cell lysis (oncotic necrosis) or programmed cellular resorption (apoptosis) depending on other factors, such as ATP [29]. In our study FABP1 attenuated the increased LDH release. It is not clear on the exact mechanism (direct or indirect) for the FABP1’s hepatoprotective effect but we speculate that it may in part be likely due to its antioxidant role.

## Conclusions

In summary, this study demonstrated that FABP1 plays an important protective function in AAP induced toxicity. The exact mechanism, however, is yet to be elucidated. Since oxidative stress is a major mitigating factor for cellular dysfunction in many diseases or drug induced complications, it seems logical that FABP1 levels could be increased to help combat the released ROS levels.

## Competing interests

The authors declare that they have no competing interests.

## Authors’ contributions

YG participated in study design, carried out the cell culture work and drafted the manuscript. YC performed some of the fluorescence studies and helped in the drafting of the manuscript. JY helped in the design of the studies. GW, YG, and FJB were instrumental in conceiving the study design, obtaining the necessary research funds to carry out the work. All authors read and approved the final manuscript.

## Pre-publication history

The pre-publication history for this paper can be accessed here:

http://www.biomedcentral.com/1471-230X/14/44/prepub
